# Advancing construction safety: YOLOv8-CGS helmet detection model

**DOI:** 10.1371/journal.pone.0321713

**Published:** 2025-05-20

**Authors:** Zhihui Wu, Xiaojia Lei, Munish Kumar

**Affiliations:** 1 Department of Civil Engineering, Anhui Communications Vocational & Technical College, Hefei, China; 2 School of Petrochemical Engineering, Hunan Petrochemical Vocational and Technical College, Yueyang, China; 3 Maharaja Ranjit Singh Punjab Technical University, Punjab, India; Sichuan University, CHINA

## Abstract

In the context of construction site safety management, real-time object detection is crucial for ensuring workers’ safety through accurate detection of safety helmets. However, traditional object detection methods often face numerous challenges in complex construction environments, such as low light, occlusion, and the diverse shapes of helmets. To address these issues, we propose an improved helmet detection model, YOLOv8-CGS, which is based on the YOLOv8 architecture and integrates optimization modules such as CBAM (Convolutional Block Attention Module), GAM (Global Attention Mechanism), and SLOU (Smooth Labeling Loss Function). The goal is to enhance the model’s detection accuracy and robustness in complex scenarios. Specifically, GAM improves the model’s attention to key regions, CBAM enhances its ability to perceive important features, and SLOU optimizes the accuracy of bounding box predictions, particularly in complex and occluded environments. Experimental results show that YOLOv8-CGS achieves accuracy rates of 94.58% and 92.38% on the SHD and SHWD datasets, respectively, which represent improvements of 5.9% and 5.94% compared to YOLOv8. This enhancement allows YOLOv8-CGS to provide more efficient and accurate helmet detection in practical applications, significantly improving the real-time monitoring capabilities for construction site safety management.

## 1 Introduction

Construction sites are high-risk work areas where potential dangers are omnipresent, ranging from falling objects from heights, and mechanical operation errors, to electrical fires—each of which can pose a serious threat to workers’ safety. As a result, site safety regulations mandate that every worker must wear a hard hat within the construction zone as the most basic form of personal protective equipment. However, relying solely on workers’ self-protection is insufficient to fully address the complex and variable hazards [1]. Therefore, an effective safety management system becomes key to ensuring the safety of the construction site. This system includes not only regular safety education and training but also encompasses real-time safety supervision and emergency response measures. Against this backdrop, the development of object detection algorithms becomes particularly significant. These algorithms provide innovative technical support for safety management by automatically identifying and locating personnel and safety equipment, such as hard hats, within the construction site, significantly enhancing the efficiency of safety monitoring and potentially preventing the occurrence of accidents [2].

However, in practical applications, this technology still faces several major issues. Firstly, various environmental conditions, such as insufficient lighting, obstructions, and the various colors and shapes of safety helmets, can affect the accuracy of detection. Secondly, object detection algorithms need to strike a balance between detection speed and accuracy, which is especially critical in dynamic and cluttered construction environments where rapid identification can be life-saving [3]. In addition, algorithms must be robust enough to handle the wide range of scenarios encountered on different construction sites, which require high adaptability and strong generalization capabilities of algorithms.

In the field of construction safety, a series of cutting-edge object detection models have been successively adopted, each enhancing accuracy and efficiency [4–7]. YOLOv3 laid the groundwork for real-time safety equipment monitoring with its rapid detection speeds, though it sometimes faced challenges in detecting small objects, such as safety helmets at a distance [8]. The advent of YOLOv5 brought significant performance improvements and accuracy enhancements, with its optimized architecture being more adept at handling scale variations of safety helmets at different distances. The DEtection TRansformer (DETR) model introduced an attention-based mechanism, eliminating the need for many hand-engineered components, and proposed a novel approach to object detection more aligned with the global context of an image, improving the recognition of safety helmets in cluttered scenes. However, the initial versions of DETR required longer training times and substantial computational resources [9]. Conditional DETR was built upon the transformer-based architecture of its predecessor, accelerating convergence and reducing training time, while maintaining the benefits of global context understanding. It showed promising results in distinguishing safety helmets from other objects with similar features [10]. DAB-DETR, an enhanced version of DETR, incorporated Deformable Attention Blocks, allowing the model to focus on a small set of key points around objects. This made it more efficient in identifying safety helmets of various shapes and those partially obscured [11]. Each of these models has contributed to the field, improving the accuracy and speed of safety helmet detection. Although each model has its strengths, continuous advancements are being made to address their respective limitations [12,13].

In response to the limitations identified in previous models for helmet detection, we propose a new improved model: YOLOv8-CGS. This model builds upon the network architecture of YOLOv8, incorporating a GAM and a CBAM, aimed at enhancing the performance of its backbone and neck networks. CBAM is cleverly integrated into the CSP module, thereby augmenting the model’s ability to capture key features of helmets. Additionally, we have adopted the latest bounding box loss function, SIoU, which not only improves the precision of bounding box localization but also optimizes the model’s performance in handling complex and occluded scenes. These innovative aspects of YOLOv8-CGS, collectively contributing to the overall architecture of the model, provide a more accurate and robust solution for effective helmet detection.

- This paper delineates the inaugural presentation of the YOLOv8-CGS model, a sophisticated object detection framework that incorporates GAM, CBAM, and SIoU. This synergetic integration augments the model’s proficiency in identifying safety helmets within multifaceted environments, simultaneously refining detection accuracy amidst occlusion and fluctuating lighting conditions.
- The YOLOv8-CGS model is optimized for the practical needs of construction site scenarios, particularly demonstrating significant improvements in safety helmet detection. This advancement holds considerable practical value in enhancing safety management at construction sites and reducing the risk of safety incidents.
- By integrating GAM, CBAM, and SIoU, the YOLOv8-CGS model achieves notable enhancements in processing speed and accuracy. Its performance in dynamic and diverse environments, in particular, marks a significant technological breakthrough for real-time safety monitoring systems, laying a reliable technical foundation for future intelligent safety surveillance systems.

The structure of this paper is organized as follows: Sect 2 introduces related work, mainly discussing the research progress of Two-Stage and One-Stage methods in object detection; Sect 3 provides a detailed explanation of the principles and implementation of the proposed YOLOv8-CGS method; Sect 4 presents the experimental results, including comparative and ablation studies; Sect 5 concludes the paper, summarizing the research findings and outlining potential future research directions.

## 2 Related work

### 2.1 Target detection using a two-stage approach

In the field of building site safety management, target detection is of paramount importance, and the two-stage method is one of the common strategies for target detection [14]. In this field, there are several related target detection models, each with its unique advantages and drawbacks. Firstly, RCNN (Region-based Convolutional Neural Network) is one of the early target detection models, which achieves high detection accuracy by selecting candidate regions and subjecting each region to convolutional neural network processing [15]. However, RCNN has relatively slow training and inference speeds, as well as a complex processing workflow. Next, SPPNet introduces spatial pyramid pooling, improving adaptability to targets of different scales, but still faces challenges in terms of performance and speed; Fast RCNN enhances the speed and efficiency of RCNN, but still requires two independent modules for training. RCNN improves the speed and efficiency of RCNN, but still requires two independent modules for training, while Faster RCNN introduces a regional proposal network, further improving speed and accuracy, although with a complex training process [16]. Finally, Cascade RCNN adopts a cascading structure, enhancing the robustness of the detector but increasing computational complexity [17]. These models provide various approaches to solving the target detection problem in construction site safety management, but each has its performance and efficiency challenges, making model selection dependent on specific application scenarios.

Despite the achievements of the two-stage method in target detection, it also exhibits some notable shortcomings. One of the most significant drawbacks is its multistage processing workflow. In the two-stage method, candidate regions need to be generated first, followed by target detection in these regions [18]. This requires two independent steps, resulting in additional computational overhead and complexity. This not only diminishes real-time detection, especially in dynamic and complex construction environments, where rapid target identification is crucial to ensuring worker safety.

### 2.2 Target detection using a one-stage approach

In object detection, one-stage methods are an important research topic, differing from two-stage methods in that they aim to achieve object detection through a single network stage [19]. For example, SSD is a fast and efficient one-stage object detection model capable of detecting multiple objects simultaneously within a single convolutional neural network, offering lower computational overhead. However, it may exhibit slightly lower performance when dealing with small objects and complex backgrounds [20]. Furthermore, YOLOv3 constitutes a rigorously optimized one-stage object detection paradigm, distinguished by its rapid inference capabilities and elevated detection precision [21]. It can handle multi-scale objects and multiclass detection, but still requires improvements in small object detection and adaptability to complex scenes. Furthermore, YOLOv4, while inheriting the attributes of rapid inference speed and high precision, necessitates further refinement in the detection of diminutive objects [22]. Moreover, YOLOv5 is another high-performance one-stage object detection model with great potential for real-time applications and large-scale datasets, but may face challenges in specific scenarios [23]. Lastly, YOLOv8 perpetuates the advancements of the YOLO series by striving to harmonize detection accuracy with processing speed, yet it requires further refinement in complex contexts. These models offer various approaches to address the one-stage object detection problem, but each comes with performance and efficiency challenges, necessitating the selection of the most suitable model based on specific application scenarios.

### 2.3 Applications of object detection algorithms

Object detection algorithms have achieved significant success across various fields. First, MIFNet (Multidimensional Information Fusion Network) [24] proposes an algorithm that improves small target detection accuracy through a multidimensional information fusion module, primarily applied in maritime target detection. This method uses an attention mechanism to fuse semantic and edge information, enhancing target positioning accuracy. However, despite significant improvements in accuracy, its performance in complex backgrounds and high-noise environments remains limited, particularly in dynamic maritime scenes. Next, in road safety, YOLOv3 [25] is used to detect objects on the road and combined with steering angle predictions to help drivers identify nearby vehicles and pedestrians. Although this method performs well in low-cost and nighttime environments, its distance estimation of objects depends on their height, which may cause errors at varying distances and angles. As a result, it has limited adaptability to dynamic road scenarios. In the field of driving assistance systems (DAS) [26], deep learning combined with sensor data is used for real-time steering angle prediction and automated adjustment. While this system enhances driving efficiency, its heavy reliance on sensors and hardware, along with the system’s complexity and cost, limits its widespread adoption in conventional vehicles. In video content analysis, SBD (Shot Boundary Detection) [27] introduces a fast video processing method based on frame active areas and separable moments to reduce computational costs and improve accuracy. While this approach improves computational efficiency and accuracy, its performance remains limited in high-dynamic video scenes, especially when rapid scene transitions occur, leading to potential misjudgments. Furthermore, the YOLO series models, such as YOLOv10 [28] and YOLOv11 [29], enhance object detection performance by optimizing model architecture and reducing computational redundancy. YOLOv10 introduces an NMS-free (Non-Maximum Suppression-free) training method to reduce inference latency, achieving a good balance between performance and efficiency. However, these models still require further optimization between accuracy and real-time performance when dealing with high-precision and multi-object complex scenarios. Finally, DETR (Detection Transformer) [30] adopts an end-to-end training method but suffers from low training efficiency due to the lack of consistent distillation points. To address this, KD-DETR [31] introduces a new knowledge distillation method, improving model performance through consistent distillation point sampling. Despite some improvements, this method still faces performance bottlenecks in high-complexity scenarios, especially when applied to large-scale datasets.

We propose YOLOv8-CGS, specifically designed for construction site safety management, aimed at addressing object detection challenges in complex environments. The model significantly improves detection accuracy and robustness by integrating optimization modules such as CBAM, GAM, and SLOU, particularly enhancing the recognition of helmets and other safety equipment under dynamic, occluded, and complex lighting conditions.

## 3 Method

### 3.1 Overview of our network

**Fig 1:**
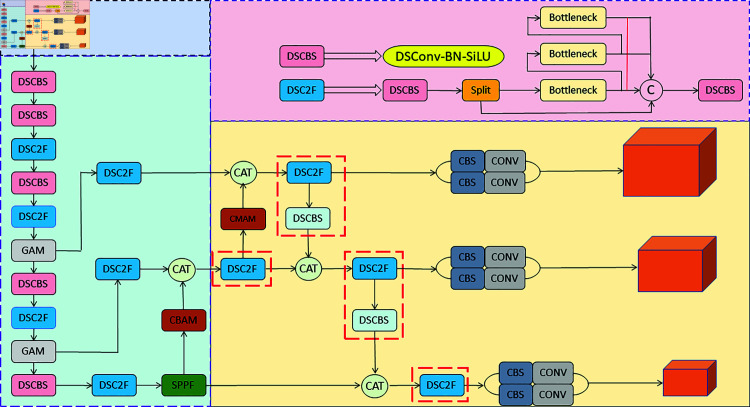
YOLOv8-CGS network structure diagram.

We have introduced an innovative object detection model named YOLOv8-CGS. This model is an enhancement based on the YOLOv8 network architecture, incorporating three key components: the GAM, the CBAM, and the latest SIoU loss. Firstly, YOLOv8-CGS introduces the GAM, which equips the model with the ability to globally focus on crucial regions when processing input images, thereby improving the accuracy of object detection. The Global Attention Mechanism allows the model to better capture contextual information about objects, contributing to enhanced detection performance. Secondly, the model adopts the CBAM, which enhances the model’s focus on object regions without increasing the network depth. CBAM achieves this by adaptively adjusting channel weights and spatial weights, enabling the model to concentrate more on critical object features, thus improving robustness and accuracy in detection. Lastly, to further optimize the training process, we have incorporated the latest SIoU loss function. This loss function provides a more precise measurement of predicted bounding boxes, thereby contributing to the refinement of the model’s performance and robustness in object detection endeavors. Furthermore, the improvements made by the YOLOv8-CGS model are closely related to improving the accuracy of helmet safety detection. Ensuring the safety of workers’ heads through the accurate detection of safety helmets is crucial in construction site safety management. The use of GAM and CBAM in YOLOv8-CGS aims to improve focus on head regions, enhancing the accuracy of safety helmet detection. The model architecture diagram of YOLOv8-CGS is depicted in Fig 1.

In the field of safety helmet detection, various complex scenarios and challenges exist, such as low lighting conditions and the diversity of safety helmet colors and shapes. Hence, the SIoU loss function introduced by YOLOv8-CGS can assist the model in more accurately measuring the position and shape of safety helmets, thus increasing the accuracy and robustness of detection. This comprehensive model enhancement is expected to bring significant technological advancements and application potential to the fields of object detection and safety helmet detection.

### 3.2 YOLOv8

**Fig 2:**
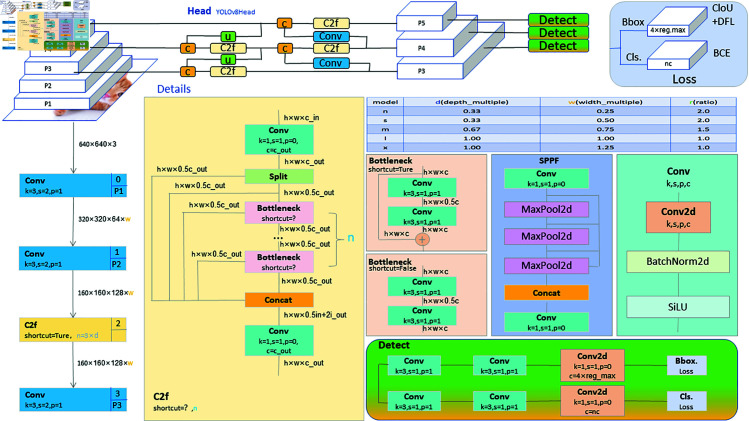
YOLOv8 network structure diagram.

YOLOv8 represents a seminal advancement in object detection models. As the most recent iteration in the YOLO series, it has garnered significant success and recognition within the object detection domain [32]. Firstly, YOLOv8 epitomizes a single-stage object detection model distinguished by its remarkable inference velocity. In the context of construction site safety management, real-time object detection is paramount to ensuring workers’ safety. The high speed of YOLOv8 makes it an ideal choice for real-time monitoring and responding to potential hazards at construction sites, thus contributing to an improved level of worker safety. Secondly, YOLOv8 excels in accuracy. Precision in object detection tasks such as safety helmet detection is crucial to ensure that workers wear helmets and that head regions are accurately identified. YOLOv8’s high accuracy effectively addresses complex detection scenarios and different colors and shapes of safety helmets, providing reliable technical support for construction site safety management. Furthermore, YOLOv8 is known for its flexibility and adaptability to various application scenarios. Construction site safety management may encounter various environmental conditions such as low lighting or obstructions. YOLOv8’s robust detection capabilities enable it to handle these challenges, ensuring worker safety. Fig 2 shows the YOLOv8 architecture.

### 3.3 Global attention mechanism

The GAM is an integral component integrated into the YOLOv8-CGS model, contributing significantly to its performance enhancement [33]. GAM’s design is aimed at addressing the challenge of capturing important contextual information across the entire input image, making it particularly relevant to our research theme, construction site safety management, and safety helmet detection.

**Fig 3:**
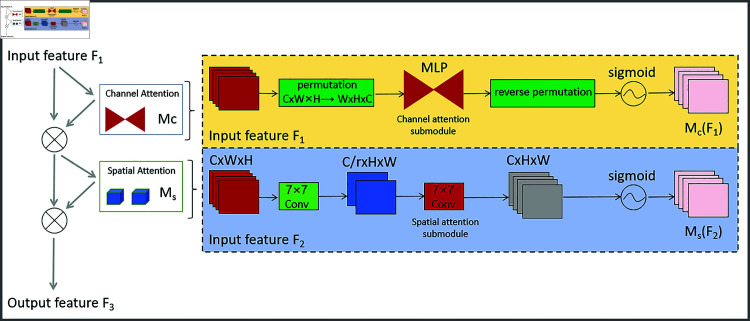
GAM network structure diagram.

GAM enables YOLOv8-CGS to focus its attention on the most critical regions of the image, which is crucial for accurate object detection. In the context of construction sites, where potential hazards and safety equipment, such as safety helmets, are distributed throughout different parts of the scene, capturing global contextual information becomes paramount. GAM achieves this by allowing the model to allocate attention to different regions of the image based on their significance, ensuring accurate detection of objects, including safety helmets, regardless of their location within the image. With GAM, YOLOv8-CGS boosts its recognition of safety helmets, even in challenging conditions like low lighting or complex environments. GAM improves the accuracy and robustness of the model, ensuring the safety of construction site workers. The architecture diagram of the GAM network is illustrated in Fig 3.

The global attention weight at position *i* is calculated as:

$$A_{i}=\frac{1}{N}\sum_{j=1}^{N}f(I_{j})$$ (1)

where: *A*_*i*_ represents the global attention weight at position *i*. *I*_*j*_ denotes the pixel value at position *j*. f(·) is the function that computes attention weights for each pixel position.

The attention weight at position *j* is determined as:

$$f(I_{j})=\frac{\exp(I_{j})}{\sum_{k=1}^{N}\exp(I_{k})}$$ (2)

where: *f*(*I*_*j*_) represents the attention weight at position *j*. exp(·) denotes the exponential function. $\sum_{k=1}^{N}\exp(I_{k})$ is the sum of exponential functions computed for all pixel positions, used to normalize the attention weights.

The global context weight at position *i* is computed as:

$$G_{i}=\frac{1}{N}\sum_{j=1}^{N}g(I_{j})$$ (3)

where: *G*_*i*_ represents the global context weight at position *i*. *I*_*j*_ denotes the pixel value at position *j*. g(·) is the function that computes global context weights for each pixel position.

The global context weight at position *j* is calculated as:

$$g(I_{j})=\frac{\exp(I_{j})}{\sum_{k=1}^{N}\exp(I_{k})}$$ (4)

where: *g*(*I*_*j*_) represents the global context weight at position *j*. exp(·) denotes the exponential function. $\sum_{k=1}^{N}\exp(I_{k})$ is the sum of exponential functions computed for all pixel positions, used to normalize the global context weights.

The final attention weight at position *i* is given by:

$$\alpha_{i}=\frac{A_{i}}{G_{i}}$$ (5)

where: $\alpha_{i}$ represents the final attention weight at position *i*. *A*_*i*_ is the weight of global attention at position *i*. *G*_*i*_ is the weight of the global context at position *i*.

### 3.4 Convolutional block attention module

**Fig 4:**
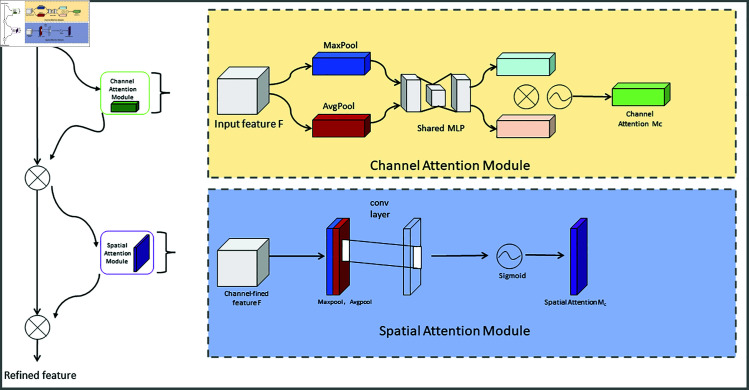
CBAM network structure diagram.

The CBAM is a feature enhancement mechanism designed to improve the performance of CNNs [34]. It encapsulates attentional information spanning both the channel and spatial dimensions of the input feature maps, thus enabling the network to accentuate the most salient features while diminishing the importance of superfluous ones. In our model, YOLOv8-CGS, CBAM is integrated and plays a critical role in improving object detection performance, particularly in safety helmet detection. The inclusion of CBAM enables YOLOv8-CGS to adaptively highlight relevant features in complex and cluttered scenes, which is essential in the context of safety management at construction sites. It helps YOLOv8-CGS concentrate on crucial aspects of the image, such as safety helmets or potential hazards, while filtering out noise and irrelevant details, thereby enhancing detection accuracy and robustness. The architecture of the CBAM network is depicted in Fig 4.

The channel-wise attention map *M*_*c*_ is computed as:

$$M_{c}=\max_{H,W}\left(\sigma (\text{MLP}(X))\right)$$ (6)

where: *M*_*c*_ denotes the channel-wise attention map. *H* and *W* signify the spatial dimensions of the feature map. σ(·) represents the activation function of the sigmoid. MLP(·) stands for a multilayer perceptron applied to the input feature map *X*.

The spatial-wise attention map *M*_*s*_ is computed as:

$$M_{s}=\text{mean}(\sigma (\text{MLP}(X)),\text{axis}=1)$$ (7)

where: *M*_*s*_ signifies the spatial-wise attention map, σ(·) designates the sigmoid activation function, MLP(·) denotes the multilayer perceptron applied to the input feature map *X*, and ‘axis=1‘ indicates the channel dimension along which the mean is calculated.

The final attention map *M* is given by:

$$M=M_{c}⊗M_{s}$$ (8)

where: *M* represents the resultant attention map. *M*_*c*_ signifies the channel-specific attention map. *M*_*s*_ denotes the spatial-specific attention map. ⊗ indicates the element-wise multiplication operation.

The refined feature map *Y* is calculated as:

Y=X⊗M (9)

where: *Y* denotes the refined feature map, *X* signifies the input feature map, *M* represents the final attention map, and ⊗ indicates element-wise multiplication.

### 3.5 SloU loss

The SIoU loss function represents an advanced metric commonly used in object detection tasks to quantify the congruence between predicted bounding boxes and their corresponding ground-truth counterparts [35]. It addresses multiple challenges posed by the traditional Intersection over Union (IoU) loss, making it particularly valuable in domains such as safety helmet detection. In our YOLOv8-CGS model, the SIoU loss offers several advantages. Firstly, it takes into account the scale variations of objects, ensuring that smaller objects are not heavily penalized while maintaining sensitivity to larger objects. This is crucial in the detection of safety helmets, where helmet sizes may vary depending on their distance from the camera. Secondly, the SIoU loss encourages precise predictions of both bounding-box coordinates and object scales. This precision is essential for accurately locating objects, such as safety helmets, as inaccurate bounding-box coordinates could lead to false positives or false negatives. Additionally, SIoU loss helps alleviate issues related to class imbalance and variations in object scales. In the field of construction site safety management, the number of safety helmets in different scenes may vary. The SIoU loss provides a balanced measure of object detection performance, assisting the model in adapting to such scenarios.

The Intersection over Union (IoU) is computed as:

$$IoU(P,G)=\frac{\text{Area of Intersection}(P,G)}{\text{Area of Union}(P,G)}$$ (10)

where: *IoU*(*P*,*G*) is the Intersection over Union of predicted box *P* and ground truth box *G*. extAreaofIntersection(P,G) is the intersection area. extAreaofUnion(P,G) is the union area.

The Scale-Invariant Intersection over Union (SIoU) is computed as:

$$SIoU(P,G)=\frac{IoU(P,G)}{1+{\text{Distance}}^{2}(P,G)}$$ (11)

where: *SIoU*(*P*,*G*) is the Scale-Invariant Intersection over Union. *IoU*(*P*,*G*) is the intersection over the union. *extDistance*^2^(*P*,*G*) is the squared Euclidean distance between the centers of *P* and *G*.

The SIoU loss *L*_*SIoU*_ is calculated as:

$$L_{SIoU}(P,G)=1-SIoU(P,G)$$ (12)

where *L*_*SIoU*_(*P*,*G*) is the SIoU loss between predicted box *P* and ground truth *G*, and *SIoU*(*P*,*G*) is the Scale-Invariant Intersection over Union.

## 4 Experiment

### 4.1 Datasets

This experiment utilizes the Safety Helmet Datasets (SHD) [36] and Safety Helmet Wearing Dataset (SHWD) [37], which have been specifically designed to address workplace safety management concerns. These datasets offer extensive image data for developing computer vision models to monitor real-time safety helmet usage. They encompass image samples from various work scenarios and environmental conditions, enhancing the robustness and generalization capabilities of the models.

Both SHD and SHWD datasets come with detailed label information, annotating the safety helmet-wearing status of workers in each image, including wearing, not wearing, or wearing improperly. These labels serve as benchmarks for model training and performance evaluation by researchers. Furthermore, these data sets offer a variety of different scenarios and conditions, such as variations in lighting, different angles, and diverse head obstructions, to simulate the complexity of actual workplace environments.

### 4.2 Experimental environment

This experiment was carried out on a PC with the specified hardware and software environment. The computational resources, including a high-end CPU and dual NVIDIA RTX3090 GPUs, were utilized to train and evaluate the proposed YOLOv8-CGS model for safety helmet detection. The software environment consisted of Windows 10 as the operating system, Python 3.9 for coding, Matplotlib for visualization, OpenCV for image processing, and CUDA 11.3 to leverage GPU acceleration for deep learning tasks. These resources and tools provided the necessary infrastructure to perform the experiments and evaluate the model’s performance effectively. The specific settings of the experimental environment are shown in the Table 1 as follows.

**Table 1:** Experiment environment.

Component	Description
Platform	PC
CPU	Intel Core i9-9900K CPU @ 3.60GHz
GPU	NVIDIA RTX3090 Graphics Card × 2 CUDA Cores
Memory	32GB
Video Memory	11GB GDDR6
Operating System	Windows 10
Python	3.9
Matplotlib	3.3.4
OpenCV	4.5.5
CUDA	11.3

### 4.3 Evaluation metrics

We use accuracy, recall, F1 score, mean Average Precision (mAP), mAP at different IoU thresholds (mAP@[IoU]), and frame rate mean Average Precision to analyze the model’s effectiveness.

Precision measures the accuracy of positive predictions made by a model. It is the ratio of true positive predictions to total positive predictions.

$$Precision=\frac{True\;Positives}{True\;Positives+False\;Positives}$$ (13)

where: True Positives represents the number of correctly identified positive cases. False Positives represents the number of falsely identified positive cases.

Recall measures a model’s ability to identify relevant instances. It is calculated as the ratio of true positives to the total actual positives.

$$Recall=\frac{True\;Positives}{True\;Positives+False\;Negatives}$$ (14)

The F1 Score is the harmonic mean of Precision and Recall, balancing them, particularly with class imbalance.

$$F1score=\frac{2\cdot Precision\cdot Recall}{Precision+Recall}$$ (15)

mAP is a common metric in object detection and information retrieval. Averages the precisions for each class and computes their mean.

$$mAP=\frac{1}{N}\sum_{i=1}^{N}AP_{i}$$ (16)

where: *N* is the number of classes. *AP*_*i*_ is the average precision for class *i*.

### 4.4 Experimental details

Step1: Data preprocessing

Within the scope of this experiment, we conducted data preprocessing by initially purifying and standardizing the raw image data from the Safety Helmet Datasets (SHD) and SafetyHelmetWearing-Dataset (SHWD) to ensure superior image quality and consistency. Subsequently, we adjusted the image dimensions to conform with the input specifications of our model. The dataset was stratified as follows: The SHD dataset, consisting of 45,200 images, was apportioned into 70% for the training set, 15% for the validation set, and 15% for the test set. Similarly, the SHWD dataset, comprising 64,830 images, was allocated 70% for the training set, 15% for the validation set, and 15% for the test set. Specifically, the SHD dataset partitioned into 31,640 images for the training set, 6,780 images for the validation set, and 6,780 images for the test set. The SHWD training set incorporated 45,831 images, the validation set contained 9,724 images, and the test set comprised 9,724 images. This partitioning scheme assures rigorous training and evaluation of our model across diverse datasets to attain optimal performance. The precise dataset partitioning is depicted in Table 2:

**Table 2:** Dataset split for SHD and SHWD.

Dataset	Total Images	Training Set	Validation & Test Set
SHD	45,200	31,640	13,560
SHWD	64,830	45,831	19,299

Step 2: Model training: In the context of model training, several key hyperparameters were carefully configured. The learning rate was set to 0.001 to control the step size during optimization. Adam’s optimizer was used to efficiently update model weights. A batch size of 32 was chosen to process multiple data samples simultaneously, improving the efficiency of the training process. Weight decay, with a value of 0.0001, was applied to prevent overfitting and control the model’s complexity. The training process consisted of 300 epochs, ensuring an adequate number of iterations for convergence. The model architecture comprised 9136374 parameters spread across 252 layers, making it a deep neural network capable of handling complex feature extraction and object detection tasks. These hyperparameter settings were instrumental in achieving the desired performance in our experiments. The specific model parameter settings are presented in Table 3.

**Table 3:** Model parameter settings.

Parameter	Value
Learning Rate	0.001
Optimizer	Adam
Batch Size	32
Weight Decay	0.0001
Training Epochs	150
Model Parameters	9,136,374
Number of Layers	252

**Fig 5:**
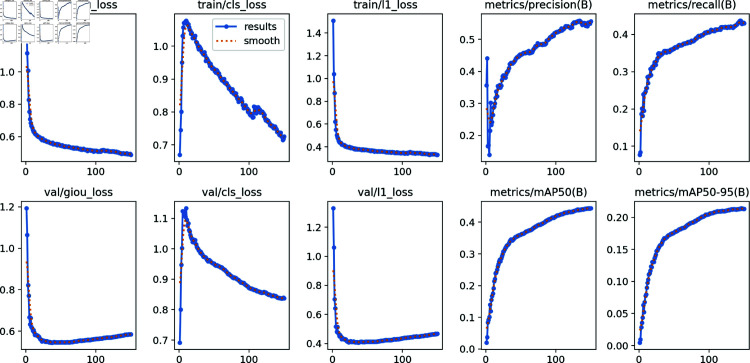
The performance of the YOLOv8-CGS model.

To ascertain the convergence of our optimized model across the datasets, we conducted a comparative analysis of critical performance metrics throughout the YOLOv8-CGS model’s training process (Fig 5). This analysis encompasses the examination of loss curves pertaining to bounding box, confidence, and class, alongside the convergence metrics of accuracy, recall, mAP@0.5, and mAP@[0.5:0.95]. These assessments facilitate the evaluation of the model’s performance trajectory during training, the identification of potential overfitting or underfitting phenomena, and the provision of optimization insights.

The algorithmic process of the YOLOv8-CGS network is shown in Algorithm 1:

**Algorithm 1. Training process for YOLOv8-CGS network.**



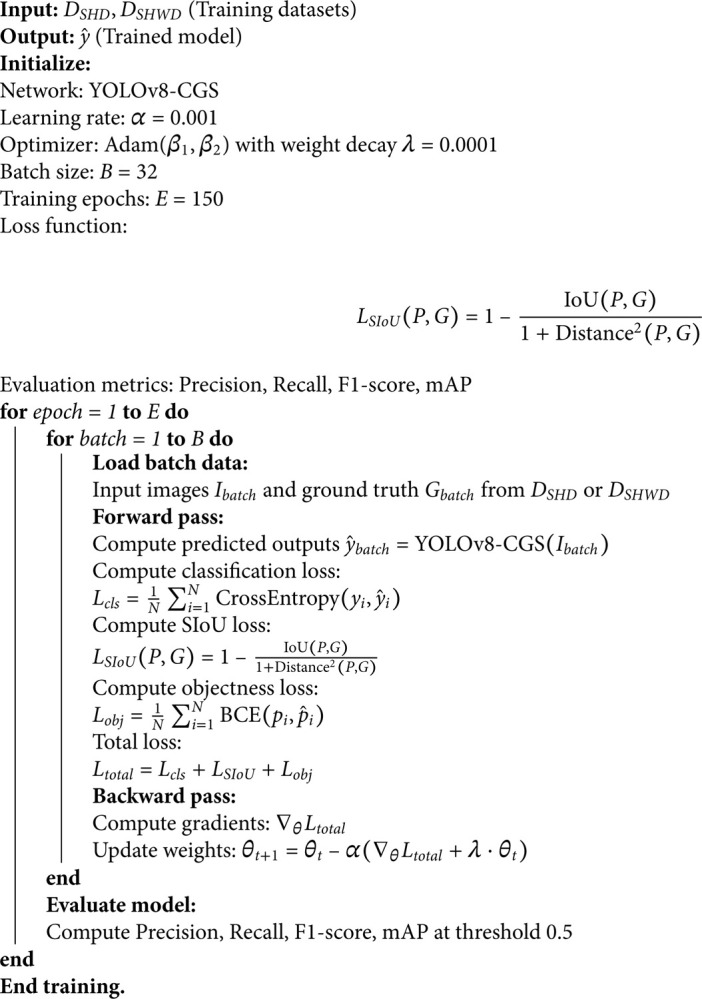



### 4.5 Experimental results and analysis

**Fig 6:**
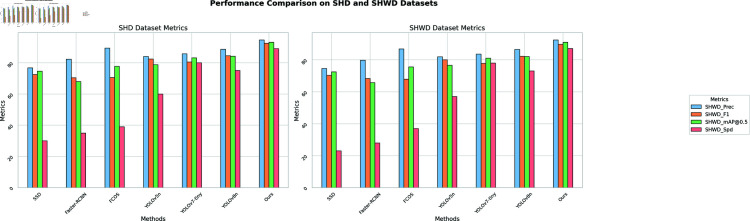
Comparison of different methods on SHD and SHWD datasets.

As shown in Table 4, our proposed method outperforms all other methods in terms of precision, F1 score, and mAP@0.5 on both SHD and SHWD datasets. Specifically, our method achieves a precision of 94. 58% and 92. 48%, a F1 score of 92.48% and 89.68%, and mAP@0.5 of 93. 18% and 90. 98% in the SHD and SHWD datasets, respectively. When comparing the detection speed, our method operates at 89 FPS, demonstrating competitive performance compared to existing methods. These results demonstrate the strong performance of our YOLOv8-CGS model in helmet detection. Our method offers significant advantages over other models, providing a more accurate and efficient solution. Fig 6, visualizing Table 4, further underscores our model’s superiority.

**Table 4:** Evaluative analysis of various methodologies applied to SHD and SHWD datasets.

Methods	SHD				SHWD			
	Prec. (%)	F1 (%)	mAP@0.5 (%)	Spd. (FPS)	Prec. (%)	F1 (%)	mAP@0.5 (%)	Spd. (FPS)
SSD [20]	76.76	72.48	74.68	30	74.58	70.25	72.45	23
Faster-RCNN [16]	82.35	70.43	67.95	35	79.76	68.28	65.74	28
FCOS [38]	89.44	70.48	77.68	39	86.72	67.84	75.49	37
YOLOv5n [39]	84.02	82.45	78.75	60	81.88	80.06	76.53	57
YOLOv7-tiny [40]	85.78	80.48	83.22	80	83.55	77.77	81.02	78
YOLOv8n [32]	88.68	84.49	84.21	75	86.44	82.22	82.08	73
Ours	94.58	92.48	93.18	89	92.38	89.68	90.98	87

As delineated in Table 5, our proposed methodology significantly outperforms in regard to both model parameters and computational complexity. In comparison to alternative models, our approach exhibits a reduced parameter count on the SHD and SHWD datasets, quantified at 5.51M and 5.31M, respectively. This diminution not only mitigates the storage demands of the model but also reduces the computational overhead. Furthermore, our method is associated with comparatively lower Floating Point Operations Per Second (FLOPs), specifically 9.66B and 9.46B for SHD and SHWD, respectively. These metrics underscore that our model sustains superior performance while optimizing computational resource utilization.

**Table 5:** Model parameters and complexity for SHD and SHWD datasets.

Model	SHD		SHWD	
	PARAMS	FLOPs	PARAMS	FLOPs
SSD	7.69M	10.66B	7.19M	9.86B
Faster-RCNN	7.50M	10.56B	6.90M	9.46B
FCOS	7.43M	10.36B	6.81M	9.26B
YOLOv5n	8.03M	10.96B	7.43M	10.16B
YOLOv7-tiny	8.23M	11.16B	7.63M	10.36B
YOLOv8n	7.93M	10.86B	7.33M	10.06B
Ours	5.51M	9.66B	5.31M	9.46B

**Fig 7:**
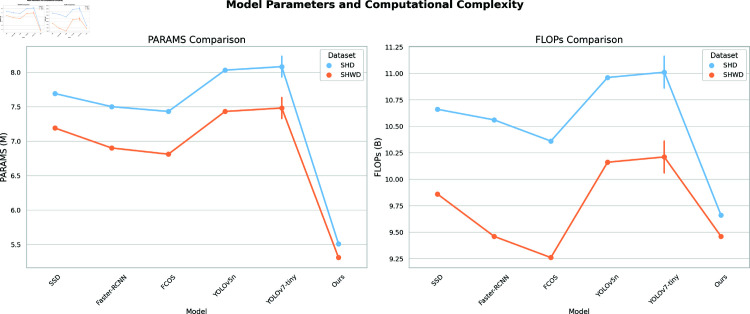
Model parameters and computational complexity for SHD and SHWD datasets.

Fig 7 delineates the tabular data, furnishing a lucid depiction of our method’s pronounced benefits with respect to model parameters and computational efficiency. This highlights our approach as a lightweight yet efficacious solution for helmet detection applications.

### 4.6 Ablation experiment

In Table 6, (1) to (5) represent results under different experimental conditions. It can be observed that, with the gradual addition of CBAM, SLOU, and GAM modules, the model performance improves incrementally. When all three modules are incorporated, our method shows outstanding performance, achieving a precision of 94. 58% and 92. 38% in the two data sets, along with the corresponding F1 scores, mAP @ 0.5, and speed metrics.The findings substantiate the efficacy of the implemented CBAM, SLOU, and GAM modules in enhancing helmet detection tasks, thereby markedly augmenting model performance.

**Table 6:** Results of ablation studies on SHD and SHWD datasets.

Method	Modules			SHD				SHWD			
	CBAM	SLOU	GAM	Precision	F1 Score	mAP	FPS	Precision	F1 Score	mAP	FPS
(1)				83.7	87.8	88.0	82	88.5	85.6	86.8	70
(2)	✓			84.72	88.4	89.9	89	86.5	86.2	87.7	81
(3)		✓		85.2	89.0	89.3	82	83.5	88.1	88.1	70
(4)	✓		✓	90.4	89.1	90.1	84	90.23	86.9	88.9	71
(5)	✓	✓	✓	94.58	92.48	93.18	89	92.38	89.68	90.98	87

## 5 Conclusion and discussion

In this research, we have introduced and developed a helmet detection model based on the YOLOv8 architecture. The efficacy of the model in helmet detection tasks was significantly improved through the integration of CBAM, SLOU, and GAM modules. Empirical evaluations demonstrated the model’s superior performance on the SHD and SHWD datasets, achieving exceptional metrics including precision (94.58%), F1 score (92.48%), mAP @ 0.5 (93.18%), and a processing speed of 89 FPS. These results highlight the potential of YOLOv8-CGS as a robust and efficient solution for real-time safety helmet detection in construction site safety management.

Despite its promising results, the proposed model has some limitations. Firstly, the dataset used for training and evaluation, while comprehensive, is still limited in its representation of diverse construction site conditions. This limitation may impact the generalizability of the model to unseen or highly complex scenarios. Secondly, the model has not undergone pruning or other optimization techniques to reduce its computational complexity further, which may hinder its deployment on edge devices or systems with limited resources.

Future research directions will focus on addressing these limitations to further enhance the model’s practicality and performance. Expanding the dataset to include more diverse and challenging construction site scenarios, such as varying weather conditions and different worker activities, will improve the model’s adaptability and robustness. Additionally, implementing model pruning and lightweight optimization techniques will enable deployment on low-power devices, facilitating broader applications in real-world environments. Through these efforts, we aim to further improve the safety management standards at construction sites, providing more reliable protection for workers’ lives.
